# High Plasma Concentration of Apolipoprotein C-III Confers an Increased Risk of Cerebral Ischemic Events on Cardiovascular Patients Anticoagulated With Warfarin

**DOI:** 10.3389/fcvm.2021.781383

**Published:** 2022-02-04

**Authors:** Oliviero Olivieri, Gianni Turcato, Manuel Cappellari, Filippo Stefanoni, Nicola Osti, Francesca Pizzolo, Simonetta Friso, Antonella Bassi, Annalisa Castagna, Nicola Martinelli

**Affiliations:** ^1^Unit of Internal Medicine, Department of Medicine, University of Verona, Verona, Italy; ^2^Department of Emergency Medicine, Franz Tappeiner Hospital, Merano, Italy; ^3^Stroke Unit, Borgo Trento Hospital, Verona, Italy; ^4^Laboratory of Clinical Chemistry and Hematology, University Hospital of Verona, Verona, Italy

**Keywords:** apolipoprotein C-III, cerebral ischemic events, anticoagulant therapy, warfarin, atrial fibrillation

## Abstract

**Introduction:**

Apolipoprotein C-III (Apo CIII) is a crucial regulator of triglyceride-rich lipoproteins (TRLs) and influences the risk of cardiovascular diseases. High levels of Apo CIII have been also associated with cerebrovascular events and earlier works showed procoagulant effects of Apo CIII. The main aim was to assess whether the plasma concentration of Apo CIII could confer an increased risk of cerebral ischemic events in anticoagulated patients at high-risk of cardioembolism.

**Methods:**

We systematically checked medical records and quantified cerebral ischemic events in a selected cohort of 118 subjects [median age 68 with interquartile range (IQR) 59–75 years, 66.9% males, 52.5% with coronary artery disease (CAD)], taking anticoagulant therapy with warfarin because of atrial fibrillation (AF) and/or mechanical prosthetic heart valves. All the subjects, enrolled between May 1999 and December 2006, were prospectively followed until death or July 31, 2018. Assessments of complete plasma lipid and apolipoprotein profiles, including Apo A-I, B, CIII, and E, were available for all subjects at enrollment.

**Results:**

After a median follow-up of 109 months (IQR, 58–187), 24 subjects (20.3%) had cerebral ischemic events: stroke (*n* = 15) and TIA (*n* = 9). Subjects with plasma concentration of Apo CIII above the median value (10.3 mg/dL) had an about three-fold increased risk of stroke/TIA than those with lower levels of Apo C-III [hazard ratio 3.08 (95%CI, 1.22–7.77)]. This result was confirmed in multiple Cox regression models adjusted for gender, age, CAD, AF, diabetes, hypertension, plasma lipids, and CHA_2_DS_2_-VASc score. By stratifying the sample on the basis of Apo CIII level and CHA_2_DS_2_-VASc score, an additive effect was observed with the highest risk in subjects with both high Apo C-III concentration and CHA_2_DS_2_-VASc score.

**Conclusion:**

High Apo CIII plasma levels may be associated with an increased risk of ischemic stroke/TIA in high-risk cardiovascular patients anticoagulated with warfarin.

## Introduction

Stroke is not only a major cause of death, but it is burdened by high morbidity with up to 50% of survivors being chronically disabled, thus having enormous health and social-economic consequences (1). Ischemic strokes account for the great majority of all types of strokes. The analysis of secular trends in ischemic stroke subtypes demonstrates that the proportion of cardioembolic stroke increased in recent years more extensively as compared with large artery stroke and small vessel stroke (2), probably as a consequence of a more intensive management of atherogenic risk factors. Notably, cardioembolic clots from heart valves or chambers are not only one of the most frequent but also a potentially preventable mechanism (3–5). Atrial fibrillation (AF) and mechanical prosthetic heart valves, as major determinants of embolic strokes, require appropriate anticoagulant prophylaxis, that is currently mandatory in the management of the patients affected by these conditions, the former if associated with an increased risk profile defined by clinical prediction rule like CHA_2_DS_2_-VASc score (3–5).

Despite being treated with anticoagulants, a substantial number of these patients still develop cardioembolic complications and are exposed to the risk of ischemic stroke and/or transient ischemic attack (TIA) (6, 7). Notably, although several factors (e.g., older age, female sex, previous stroke/TIA, previous aspirin use, high prediction rule scores, and renal impairment) have been statistically associated with such risk (8), little is known regarding the precise mechanisms involved in such residual risk and able to breach the anticoagulant protection. In this context, we recently published a prospective study in a cardiovascular cohort of 950 subjects with or without coronary artery disease (CAD) showing that high apolipoprotein CIII (Apo CIII) plasma concentration may be associated with an increased risk of ischemic stroke or transient ischemic attack (TIA) (9). The effect of Apo CIII on cerebrovascular risk appeared stronger in subjects without signs of relevant atherosclerotic disease but with valvular heart disease (9), including patients with mechanical prosthetic heart valves and/or atrial fibrillation, i.e., well recognized conditions at risk of cardioembolic stroke. Such observation prompted us to consider that Apo CIII may influence the risk of cerebrovascular events beyond atherosclerosis-related pathways. Notably, in previous works our group also showed that high Apo CIII plasma concentration is associated with a prothrombotic diathesis, characterized by enhanced thrombin generation (10), increased factor II coagulant activity (11), and higher activated factor VII-antithrombin complex (FVIIa-AT) (12).

On the basis of these premises, we hypothesized that high Apo CIII levels leading to a prothrombotic diathesis could be associated with an increased risk of ischemic stroke/TIA in the specific clinical setting of patients taking anticoagulant therapies. Therefore, in order to investigate the factors potentially contributing to cerebrovascular risk despite anticoagulation, we selected from the previous study cohort (9) the subgroup of patients who were treated with warfarin for either atrial fibrillation (AF) or mechanical prosthetic heart valves (MPHV), which are well-recognized clinical conditions at high-risk of cardioembolic events. In particular, we reassessed whether the plasma concentration of Apo CIII could still contribute to the mechanisms leading to cerebral ischemic events in cardiovascular patients taking anticoagulant therapy with warfarin.

## Materials and Methods

### Study Population

A detailed description of the original study population has already been reported in a previous work (9). This observational study was performed within the framework of the Verona Heart Study (VHS), a regional survey that assessed cardiovascular risk factors in subjects with angiographic documentation of the state of their coronary vessels (9–12). All the subjects in the VHS are required to have no history of any acute illness in the month preceding the enrollment. CAD patients with acute coronary syndromes were excluded from this study. Subjects with severe renal failure (estimated glomerular filtration rate <30 mL/min) and those with severe hepatic impairment (clinically defined diagnosis of liver cirrhosis) were also excluded from this study. Briefly, in the original analysis (9) a total of 950 subjects enrolled between May 1999 and December 2006 with angiographic documentation of their coronary vessels, who lived in the District of Verona and for whom both prospective data on cerebrovascular events and laboratory data on baseline plasma lipids were available, were included in the original analysis. Among those patients we selected all the 118 subjects who were taking anticoagulant therapy with warfarin, because of AF and/or MPHV (*n* = 62 and *n* = 56, respectively), i.e., clinical conditions at high-risk of cardioembolic events. All subjects had a complete routinary laboratory evaluation, including Apo A-I, Apo B, Apo CIII and Apo E plasma concentrations (for analytical methods, see ref. (9)). CHA_2_DS_2_-VASc score was calculated according to the ACC/AHA/HRS 2019 guidelines (13).

### Assessment of Outcome

The subjects were followed until death or July 31, 2018. Follow-up data were available for all the subjects in the sample of the present analysis. Study subjects' mortality status was determined by searching in the National Population Register. Survival times were calculated starting from the date of enrolment. The electronic medical records of all the Hospitals in the District of Verona, Northeast Italy, including data of Emergency Units admissions were obtained for all subjects; ambulatory or telephone survey was performed in case of clinical doubt. In order to be included in the statistical computation, non-fatal ischemic stroke/TIA events had to be confirmed and validated by two independent neurologists after careful evaluation of all the available information (9). Ischemic stroke was defined as a focal neurological deficit with acute onset and presence of a corresponding lesion on diffusion weighted magnetic resonance imaging [DWI] or, if no MRI was acquired, signs of early ischemic injury on CT (14). TIA was defined as an acute onset focal neurological deficit of presumed ischemic origin without a corresponding lesion on DWI or, if no MRI was acquired, lasting <24 h (15). According to the Trial Org 10172 in Acute Stroke Treatment (TOAST) classification, etiologic ischemic stroke/TIA subtypes were the following: (1) large-artery atherosclerosis, (2) cardioembolism, (3) small-vessel occlusion, (4) stroke of other determined etiology, and (5) stroke of undetermined etiology (15). Diagnoses are based on clinical features and on data collected by tests such as brain imaging (CT/MRI), cardiac imaging (echocardiography, etc.), duplex imaging of extracranial arteries, arteriography, and laboratory assessments for a prothrombotic state.

### Statistical Analysis

Statistical analyses were performed using the SPSS 23.0 (SPSS Inc., Chicago, IL, USA) and STATA 13.0 (StataCorp, College Station, TX, USA) statistical packages. Distributions of continuous variables were expressed as median value with interquartile range (IQR). Categorical variables were expressed as proportions. Quantitative data distributions were assessed using Mann-Whitney test and the comparisons were presented also as median difference with 95% confidence interval (CI). Qualitative data were analyzed by χ^2^ test or χ^2^ for linear trend analysis when indicated. Ischemic stroke/TIA event rates during the follow-up period were assessed by using the Kaplan–Meier method with Log-rank statistic and Cox regression. Kaplan–Meier curves were used for survival plots, which stratified the study population according to Apo CIII plasma concentration. Taking into account the well-recognized role of CHA_2_DS_2_-VASc score in predicting the risk of cardioembolic events [e.g., it is a codified clinical prediction rule for estimating the risk of stroke in subjects with AF (13)], we stratified the whole study sample by Kaplan-Meier curves for ischemic stroke/TIA events according to both Apo CIII levels and CHA_2_DS_2_-VASc score. Multivariate Cox proportional models for ischemic stroke/TIA events were performed considering the Apo CIII median value (10.3 mg/dL) as threshold and including in the different models potential confounding factors, like sex, age, CAD diagnosis, atrial fibrillation, diabetes, hypertension, all plasma lipid parameters, and CHA_2_DS_2_-VASc score. Considering the high number of variables in this analysis, a final model with backward stepwise selection of variables was performed with *P* > 0.10 as the critical value for excluding variables in the model. Subjects with missing data were excluded from multivariate analysis in Cox regression models. Hazard ratios (HRs) and 95% CIs are reported with two-tailed probability values. A value of P < 0.05 was considered statistically significant.

## Result

After a median follow-up of 109 months (IQR, 58–187), 24 subjects (20.3%) had non-fatal ischemic stroke (*n* = 15) or TIA (*n* = 9) events despite the reported anticoagulant treatment. In 23 out of 24 subjects the cerebral ischemic events were classified as cardioembolic. Clinical and laboratory characteristics of the study population, as a whole and divided on the basis of ischemic stroke/TIA events during the follow-up, are summarized in Table 1 and Supplementary Table S1. Subjects with cerebral ischemic events had generally, although without statistical significance, higher plasma concentrations of both Apo B and Apo CIII (Table 1 and Supplementary Table S1). On the basis of the previous results in the original VHS cohort, suggesting Apo CIII levels as a predictor of cerebral ischemic events (9), we compared subjects with Apo CIII plasma concentrations below and above the median level (10.30 mg/dL), whose clinical and biochemical features are reported in Table 2 and Supplementary Table S2. Subjects with high Apo CIII levels had an increased rate of ischemic stroke/TIA during the follow-up [30.51% (18/59) vs. 10.17% (6/59), as compared with subjects with low Apo CIII levels, *P* = 0.006]. As expected, subjects with high Apo CIII levels had an unfavorable plasma lipid profile, with increased concentrations of both total and LDL cholesterol, triglycerides, Apo B and Apo E (Table 2 and Supplementary Table S2). Subjects with high Apo CIII levels had also an increased prevalence of diabetes and hypertension. When clinical history elements were assessed by CHA_2_DS_2_-VASc score, patients with higher Apo CIII had more frequently an increased score >2 (Table 2).

**Table 1:** Clinical and laboratory characteristics of the study sample at time of enrollment considered as a whole and subdivided on the basis of the occurrence of non-fatal ischemic stroke or TIA events during the follow-up.

	**All the subjects (*n* = 118)**	**No Stroke/TIA (*n* =94)**	**Stroke/TIA (*n* = 24)**
Age (years)	68 (59–75)	68 (59–75)	68 (60–76)
Male gender (*n*, %)	79, 66.95	65, 69.15	14, 58.33
BMI (kg/m^2^)	26.45 (23.23–29.88)	26.63 (23.14–29.74)	25.89 (23.37–29.95)
Coronary artery disease (*n*, %)	62, 52.54	50, 53.19	12, 50.00
Previous stroke/TIA (*n*, %)	8, 6.78	6, 6.38	2, 8.33
Congestive heart failure (*n*, %)	10, 8.47	10, 10.63	0, 0.00
Atrial fibrillation (*n*, %)	62, 52.54	50, 53.19	12, 50.00
Diabetes (*n*, %)	25, 21.18	20, 21.28	5, 20.83
Hypertension (*n*, %)	66, 55.93	51,54.26	15, 62.50
Smoke habit (*n*,%)	50, 49.15	48, 51.06	10, 41.67
**CHA**_**2**_ **DS**_**2**_ **-VASc score (** ***n*** **, %)**			
0	18, 15.25	16, 17.02	2, 8.33
1	26, 22.03	21, 22.34	5, 20.83
≥2	74, 62.71	57, 60.64	17, 70.83
**Laboratory parameters**			
Creatinine (μmol/L)	90.00 (77.70–105.03)	91.95 (79.28–105.10)	82.15 (75.72–94.21)
Total Cholesterol (mmol/L)	4.88 (4.02–5.89)	4.75 (3.98–5.72)	5.08 (4.37–5.97)
LDL Cholesterol (mmol/L)	3.15 (2.64–4.00)	3.17 (2.59–4.09)	3.12 (2.66–4.01)
HDL Cholesterol (mmol/L)	1.24 (1.04–1.46)	1.26 (1.05–1.49)	1.15 (1.01–1.38)
Triglyceride (mmol/L)	1.50 (1.10–1.98)	1.41 (1.09–1.98)	1.60 (1.13–2.03)
Apo AI (g/L)	1.32 (1.14–1.50)	1.33 (1.13–1.50)	1.27 (1.19–1.54)
Apo B (g/L)	0.95 (0.78–1.18)	0.94 (0.77–1.14)	1.09 (0.87–1.24)
Apo CIII (mg/dL)	10.30 (9.00–12.34)	9.89 (8.86–12.12)	11.51 (10.24–14.52)
Apo E (g/L)	0.036 (0.029–0.046)	0.037 (0.031–0.047)	0.034 (0.026–0.044)

*Distributions of continuous variables were expressed as median value with interquartile range (IQR). Categorical variables were expressed as proportions*.

**Table 2:** Clinical and laboratory characteristics of the study sample at time of enrollment subdivided according to Apo CIII plasma concentration with the median value (10.3 mg/dL) as threshold level.

	**Apo CIII < 10.3 mg/dL (*n* = 59)**	**Apo CIII ≥ 10.3 mg/dL (*n* = 59)**
Age (years)	67 (60–76)	69 (59–74)
Male gender (*n*, %)	47, 79.66	32, 54.24
BMI (kg/m^2^)	27.16 (23.23–28.98)	25.48 (23.19–30.15)
Coronary artery disease (*n*, %)	29, 49.15	33, 55.93
Previous stroke/TIA (*n*, %)	4, 6.78	4, 6.78
Congestive heart failure (*n*, %)	6, 10.17	4, 6.78
Atrial fibrillation (*n*, %)	29, 49.15	33, 55.93
Diabetes (*n*, %)	8, 13.5	17, 28.81
Hypertension (*n*, %)	27, 45.76	39, 66.10
Smoke habit (*n*, %)	26, 44.07	32, 54.24
**CHA**_**2**_ **DS**_**2**_ **-VASc (** ***n*** **, %)**		
0	15, 25.42	3, 5.08
1	11,18.64	15, 25.42
≥2	33, 55.93	41, 69.49
**Laboratory parameters**		
Creatinine (μmol/L)	92.40 (79.73–105.10)	86.30 (76.44–104.25)
Total Cholesterol (mmol/L)	4.47 (3.80–5.14)	5.40 (4.43–6.33)
LDL Cholesterol (mmol/L)	2.96 (2.34–3.62)	3.59 (2.76–4.35)
HDL Cholesterol (mmol/L)	1.20 (1.04–1.34)	1.26 (1.04–1.59)
Triglyceride (mmol/L)	1.13 (0.95–1.49)	1.89 (1.46–2.37)
Apo AI (g/L)	1.20 (1.02–1.42)	1.42 (1.23–1.58)
Apo B (g/L)	0.86 (0.71–1.04)	1.09 (0.94–1.32)
Apo CIII (mg/dL)	9.02 (7.92–9.68)	12.30 (11.41–15.76)
Apo E (g/L)	0.034 (0.029–0.040)	0.039 (0.032–0.049)
**Follow-up events**		
Ischemic Stroke/TIA (*n*, %)	6, 10.17	18, 30.51

*Distributions of continuous variables were expressed as median value with interquartile range (IQR). Categorical variables were expressed as proportions*.

**Figure 1:**
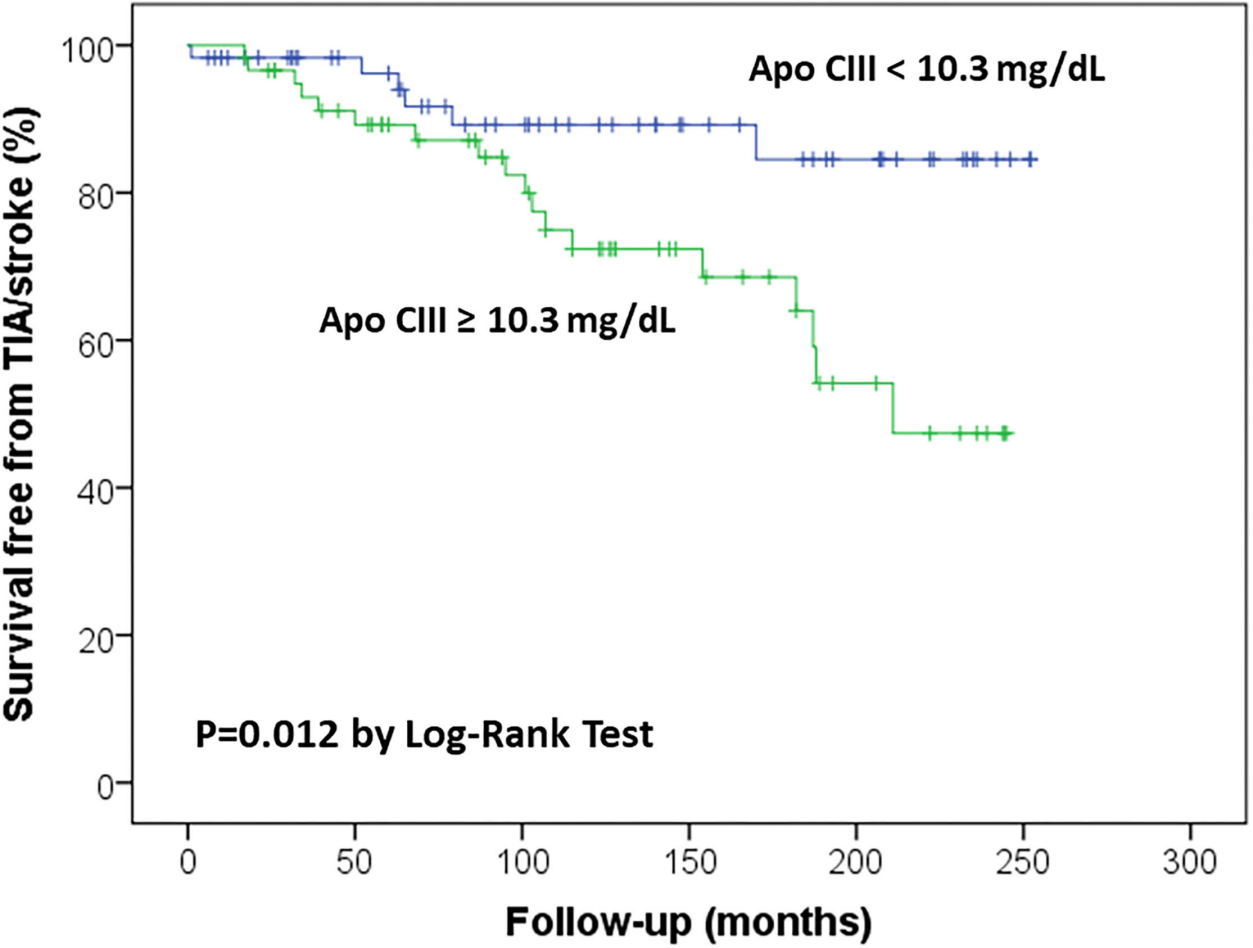
Kaplan-Meier survival curves for ischemic stroke/transient ischemic attack (TIA) in the study sample according to Apolipoprotein C-III (Apo CIII) plasma concentration, with the median level (10.3 mg/dL) as threshold value.

Kaplan-Meier survival curves confirmed that subjects with high Apo CIII levels (≥10.30 mg/dL) had an increased rate of ischemic stroke/TIA (Figure 1), with an about three-fold increased risk by Cox regression analysis as compared with those with low Apo CIII levels (Table 3). High Apo CIII levels remained associated with ischemic stroke/TIA by including progressively potential confounding factors in the regression models, like age, sex, CAD, AF, diabetes, hypertension, plasma lipids and apolipoproteins, and CHA_2_DS_2_-VASc score (Table 3), and even including all these parameters into a Cox regression model with backward stepwise selection of variables (HR 5.22 with 95%CI 1.43–19.01).

**Table 3:** Association between high plasma concentration of Apo CIII (≥10.3 mg/dL), and ischemic stroke/TIA events by different Cox Regression models (subjects with low plasma concentration of Apo CIII, <10.3 mg/dL, are considered as reference group).

	**Coefficient B**	**SE**	**Hazard ratio**
Unadjusted	1.12	0.47	3.08 (1.22–7.77)
Model 1	1.09	0.49	2.97 (1.14–7.74)
Model 2	1.05	0.49	2.86 (1.10–7.53)
Model 3	0.99	0.49	2.70 (1.03–7.13)
Model 4	1.32	0.57	3.76 (1.24–11.42)
Model 5	1.32	0.58	3.73 (1.20–11.61)

*Model 1: sex- and age-adjusted*.

*Model 2: adjusted for sex, age, CAD diagnosis, and atrial fibrillation*.

*Model 3: adjusted for sex, age, CAD diagnosis, atrial fibrillation, diabetes, and hypertension*.

*Model 4: adjusted for sex, age, CAD diagnosis, atrial fibrillation, diabetes, hypertension, and all plasma lipid parameters (i.e., total, LDL, and HDL-cholesterol, triglycerides, Apo AI, Apo B, and Apo E)*.

*Model 5: adjusted for sex, age, CAD diagnosis, atrial fibrillation, diabetes, hypertension, all plasma lipid parameters (i.e., total, LDL, and HDL-cholesterol, triglycerides, Apo AI, Apo B, and Apo E), and CHA_2_DS_2_-VASc score*.

**Figure 2:**
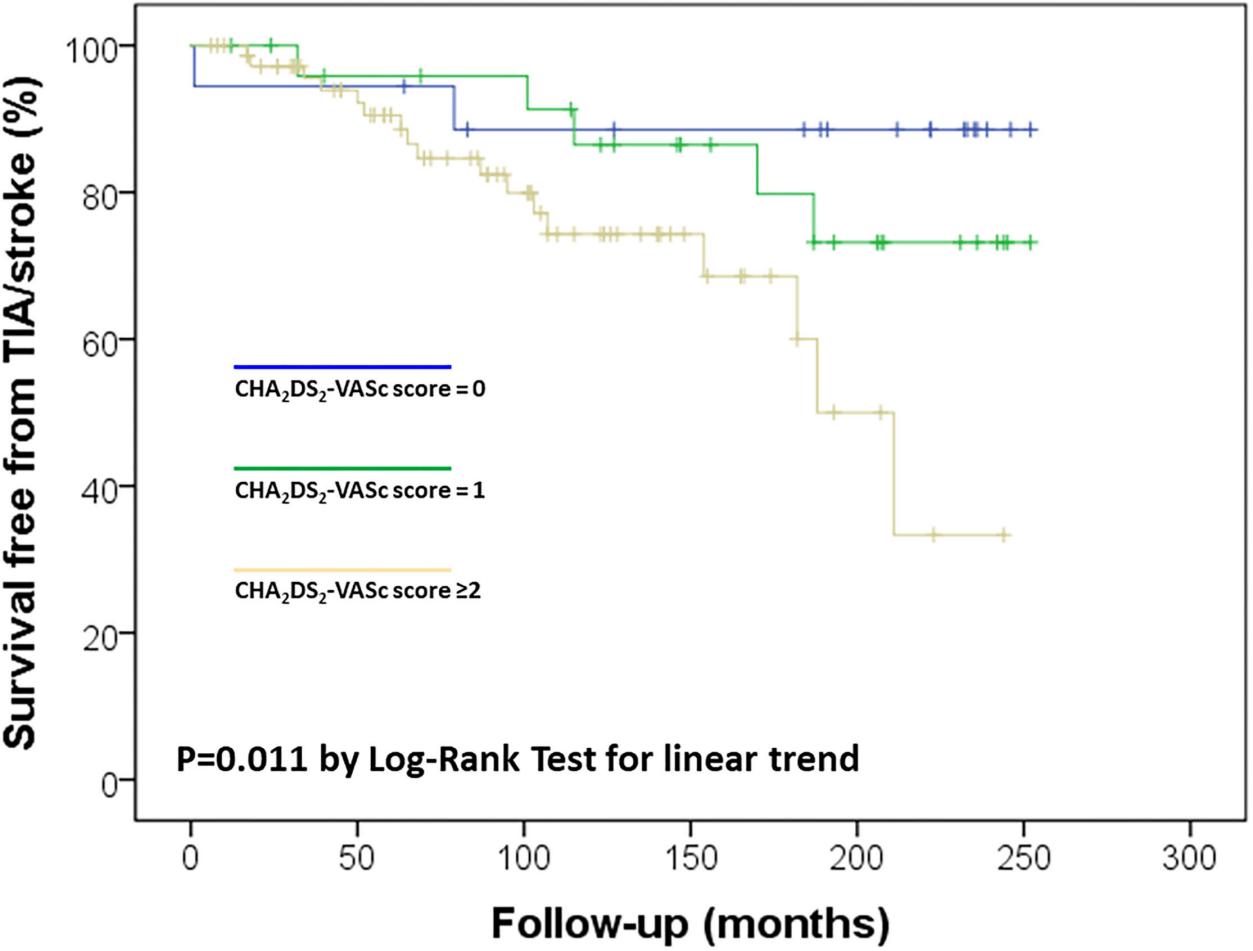
Kaplan-Meier survival curves for ischemic stroke/transient ischemic attack (TIA) in the study sample according to CHA_2_DS_2_-VASc score.

**Figure 3:**
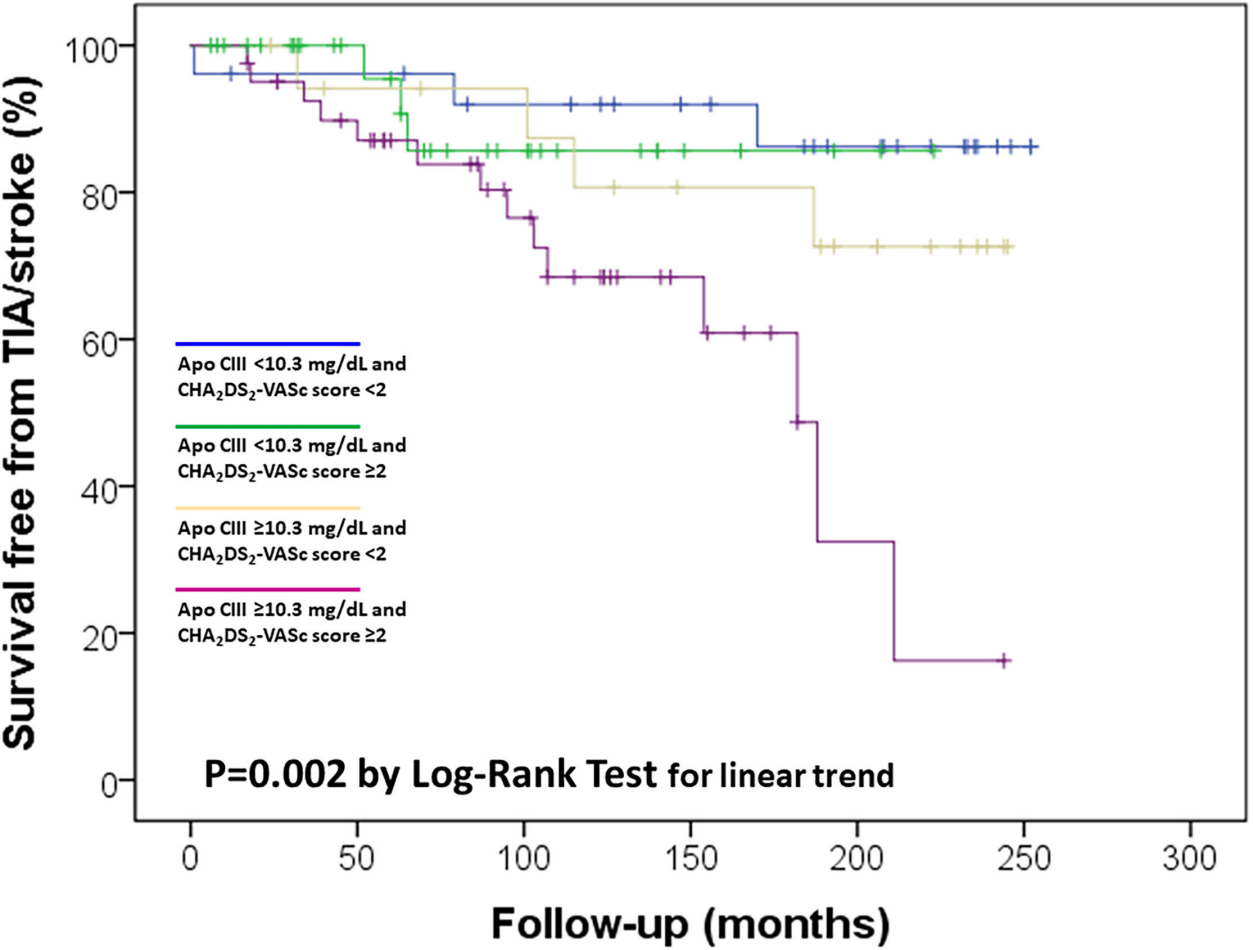
Kaplan-Meier survival curves for ischemic stroke/transient ischemic attack (TIA) in the study sample according to Apolipoprotein C-III (Apo CIII) plasma concentration, with the median level (10.3 mg/dL) as threshold value, and CHA_2_DS_2_-VASc score, with ≥2 as threshold value.

Considering the observed relation between Apo CIII levels and CHA_2_DS_2_-VASc score, which was associated with ischemic stroke/TIA in the study sample (Figure 2), a possible synergic additive effect was hypothesized. Kaplan-Meier curves were used to further dissect their association with cerebral ischemic events. We stratified the study sample in four subgroups according to the different combination of these two variables: low/high Apo CIII (threshold level at median value 10.3 mg/dL) and low/high CHA_2_DS_2_-VASc score (threshold level 2 or more points, which connote moderate to high-risk according to guidelines (13)). By this analysis, subjects with both high Apo CIII and CHA_2_DS_2_-VASc score had the highest risk of ischemic stroke/TIA as compared with those with both low Apo CIII and CHA_2_DS_2_-VASc score who had the lowest risk of cerebral ischemic events, while subjects with either low Apo CIII/high score or high Apo CIII/low score showed an intermediate risk (Figure 3).

## Discussion

As a necessary preliminary consideration, we must recognize that the current results are the by-product of a larger analysis showing that Apo CIII levels may confer an increased risk of ischemic cerebrovascular events in cardiovascular patients (8). This study was not originally designed to investigate factors influencing the residual risk of ischemic stroke in high-risk patients treated with anticoagulants. The sample size is small with consequent reduction of study power. All these issues may reduce the potential significance of our findings. Nonetheless, taking into account the proofs indicating a procoagulant effect of Apo CIII (10–12), we hypothesized that its high plasma concentration may be particularly harmful in clinical conditions requiring anticoagulation. Therefore, according to the available data, we selected a subgroup of 118 anticoagulated patients and attempted to assess whether Apo CIII could play a prognostic role in this specific clinical setting.

The findings showed that was just the case. With the limitations due to the above-mentioned premise, the present results are the first to provide evidence suggesting such a role for Apo CIII, with its high levels being associated with an about three-fold increased risk of ischemic cerebrovascular events during an about 10-year follow-up. Notably, three-quarters of subjects (18/24) with ischemic stroke/TIA during the follow-up had basal Apo CIII plasma concentrations above the median value (Table 2). Apo CIII is a crucial regulator of lipoprotein metabolism and a specific marker of TG-rich lipoproteins (TRLs). High plasma levels of Apo CIII are well-recognized to be associated with atherosclerotic diseases and inhibiting Apo CIII is now considered an attractive way of reducing not only triglyceride levels but also residual cardiovascular risk (16). Our results are consistent with this position which attributes a prominent role to Apo CIII in the setting of prevention of cardiovascular disease. Our data show that in the VHS population a substantial risk of cerebral ischemic events in the context of secondary prevention is associated with Apo CIII (and, thus, with the metabolism of TRLs), even despite anticoagulant protection. Previous reports in patients treated with vitamin K antagonists or direct oral anticoagulants (DOACs) indicated hyperlipidemia as a possible risk factor for recurrent cerebral ischemic events (6–8, 17). However, the type of hyperlipidemia was not clearly specified (6, 17) and generally emphasis was more devoted to total and LDL cholesterol rather than TG or TRLs (18). Accordingly, LDL-cholesterol-lowering treatment by statins, ezetimibe, and/or PCSK9 inhibitors is preferentially indicated for secondary prevention of stroke so far (19). However, statins are also potent Apo C III-lowering drugs (20) and their cardiovascular protective role may express by affecting both LDL and TRLs metabolism. Therefore, our present and previous results (9), suggesting a role of Apo CIII in cerebral ischemic disease, may be considered as consistent with the recommendations in current therapeutic guidelines which indicate high-dose statin (e.g., atorvastatin 80 mg), if needed also associated with ezetimibe and/or PCSK9 inhibitors, to reduce the risk of stroke recurrence (18). It should be noted that it is generally difficult to dissect the individual role of lipoproteins that share common lipid and protein-based components. In similar way, the lipid profile of our patients with high Apo CIII plasma concentration corresponded to so called “atherogenic dyslipidemia” (21, 22) that is mainly characterized by elevated TRLs levels, but also by increased Apo B, total and LDL cholesterol levels (Table 2). Nonetheless, in our study cohort among the assessed plasma lipids and apolipoproteins only Apo CIII was associated with cerebral ischemic events after adjustment for all plasma lipids, as well as for other potential confounding factors like diabetes and hypertension (Table 3).

Earlier studies identified older age, female sex, renal impairment, previous stroke/TIA, previous aspirin use, and higher CHA_2_DS_2_-VASc score as risk factors for recurrence of cerebral ischemic events (6–8, 17). In our study sample some of these parameters were not available among the collected data for analysis (e.g., previous aspirin use) and the small sample size did not allow to investigate adequately these associations. CHA_2_DS_2_-VASc score—as expression of a combination of several clinical features—was confirmed as an important risk factor, notably with an evident synergic role with Apo CIII levels (Figure 3). In other words, subjects with both atherogenic dyslipidemia marked by high Apo CIII concentrations and a clinical history coherent with an elevated risk marked by high CHA_2_DS_2_-VASc score resulted at the highest risk of ischemic stroke/TIA in spite of anticoagulant treatment, thus being potentially the “best target” for more aggressive therapeutic approaches aiming at the reduction of cerebral ischemic events. The current results also raise concerns about the prothrombotic potential triggered by high concentrations of Apo CIII, apparently potent enough to hinder the protection afforded by anticoagulation. Coagulation pathway needs lipids and high levels of cholesterol and triglyceride have been related with increased coagulation activity (23). Some previous results suggested specifically a procoagulant activity of Apo CIII in the setting of both arterial atherosclerotic vascular disease and venous thromboembolism. In a prospective study of subjects with angiographically proven CAD, we first demonstrated that thrombin generation was amplified in patients with elevated Apo CIII concentrations (10). Similarly, high levels of Apo CIII (but not other plasma lipids or apolipoproteins) were associated with a progressive increase in factor II coagulant activity (11). Apo CIII was also strongly associated with the activated FVII–antithrombin (FVIIa-AT) complex, which is an indirect marker of intravascular exposure of tissue factor (TF), thus providing suggestion for an Apo CIII-related activation of the extrinsic coagulation pathway (12). In a recent study involving 127 patients with venous thromboembolism and 299 controls, concentrations of Apo CI, CII, CIII and E were associated with several coagulation factors, including vitamin K-dependent factors, as well as factor XI, factor VIII, and von Willebrand factor levels (24). Finally, within the framework of VHS, in a cohort of 1,020 of patients with cardiovascular disease, subjects with high Apo CIII concentrations had an about three-fold increased risk to experience venous thromboembolic events within a long-term period of 12 years as compared with those with low Apo CIII levels (25). All these lines of evidence are consistent with an active interplay between TG-rich/Apo CIII-rich lipoproteins and the mechanisms of coagulation and, thereby, may represent a plausible biological ground for the results of the current analysis. Nevertheless, the present findings do not allow us to get any causal inference to why anticoagulated patients with higher levels of Apo CIII are less protected from the risk of ischemic stroke over time. Our results only show a mere statistical association between two objective parameters, i.e., apolipoprotein plasma concentration and number of cerebral ischemic events. This work has some significant limitations which should be acknowledged beyond the previously mentioned hindrances due to a sub-analysis not primarily designed for such purpose and with a small sample size. Notably, our results pertain substantially to dicumarolic anticoagulants. All the subjects of this analysis were treated with warfarin at time of enrollment between May 1999 and December 2006, but we cannot exclude for patients with AF without mechanical prosthetic heart valves the possibility of a shift to DOACs in the last period of follow-up, i.e., since April 2013 (when dabigatran became the first approved DOAC for stroke prevention in AF in Italy). Most importantly, we have not been able to control compliance to anticoagulant drugs over time. However, it is also difficult to hypothesize an asymmetric distribution of drug compliance according to Apo CIII concentration. Taking into account all these limitations, the present results provide, for the first time, an unexpected basis for suspecting a role of Apo CIII in the residual risk of stroke in patients with AF and/or mechanical prosthetic heart valves treated with anticoagulants. There is now a great need of research investigating the mechanisms of stroke recurrence and improving secondary prevention (26). If our data would be corroborated by further studies, patients with promptly identified increased Apo CIII levels could speculatively take advantage of a more intensive treatment strategy, including higher anticoagulant dosages and/or specific lipid (Apo CIII)-lowering therapies. Anyway, future investigations will be needed to fully confirm these observations and the related clinical implications, as well as to clarify the underlying molecular mechanisms.

## Data Availability Statement

The datasets presented in this article are not readily available because data cannot be shared publicly due to the privacy of individuals that participated in the study. The data will be shared on reasonable request to the corresponding author. Requests to access the datasets should be directed to oliviero.olivieri@univr.it and nicola.martinelli@univr.it.

## Ethics Statement

The studies involving human participants were reviewed and approved by Azienda Ospedaliera Universitaria Integrata of Verona, Italy. The patients/participants provided their written informed consent to participate in this study.

## Author Contributions

OO, GT, and NM contributed to conception and design of the study. FS, GT, and MC organized the database. NM, FP, and SF performed the statistical analysis. AC, NO, MC, and AB participated to data acquisition and analysis. NM and OO drafted the manuscript. All authors contributed to manuscript revision, read, and approved the submitted version.

## Funding

This work was supported by the Cariverona Foundation (project B36J16002570003). The funder was not involved in the study design, collection, analysis, interpretation of data, the writing of this article or the decision to submit it for publication.

## Conflict of Interest

MC declares consulting fees from Boehringer-Ingelheim and Pfizer-BMS, and advisory board from Daiichi Sankyo. The remaining authors declare that the research was conducted in the absence of any commercial or financial relationships that could be construed as a potential conflict of interest.

## Publisher's Note

All claims expressed in this article are solely those of the authors and do not necessarily represent those of their affiliated organizations, or those of the publisher, the editors and the reviewers. Any product that may be evaluated in this article, or claim that may be made by its manufacturer, is not guaranteed or endorsed by the publisher.
