# Supplementary material for: High Plasma Concentration of Apolipoprotein C-III Confers an Increased Risk of Cerebral Ischemic Events on Cardiovascular Patients Anticoagulated With Warfarin
Source: Front Cardiovasc Med. 2022 Feb 4;8:781383. doi: 10.3389/fcvm.2021.781383 (PMC8854278; doi:10.3389/fcvm.2021.781383)
Supplement: Supplementary file 1 [file Data_Sheet_1.docx]

**SUPPLEMENTARY MATERIAL**

**S1 Table. Comparisons between patients with or without TIA for quantitative parameters expressed by median difference with 95% confidence interval.**

|  | **Median difference with 95%CI** |
| --- | --- |
| Age (years) | 0  (-5 – 5) |
| BMI (kg/m²) | 0.48  (-1.63 – 2.21) |
| Creatinine (µmol/L) | 6.53  (-2-14.77) |
| Total Cholesterol (mmol/L) | -0.41  (-0.97-0.17) |
| LDL Cholesterol (mmol/L) | -0.08  (-0.55-0.42) |
| HDL Cholesterol (mmol/L) | 0.07  (-0.08-0.2) |
| Triglyceride (mmol/L) | -0.10  (-0.41-0.19) |
| Apo AI (g/L) | 0.01  (-0.11-0.14) |
| Apo B (g/L) | -0.12  (-0.23-0.01) |
| Apo CIII (mg/dL) | -1.34  (-2.66-0.14) |
| Apo E (g/L) | 0.005  (-0.001-0.010) |

**S2 Table. Comparison between patients presenting ApoCIII values above or below the median level of 10.3 mg/dL, for quantitative parameters expressed by median difference with 95% confidence interval.**

|  | **Median difference with 95%CI** |
| --- | --- |
| Age (years) | 1  (-3-5) |
| BMI (kg/m²) | 0.48  (-1.37-2.23) |
| Creatinine (µmol/L) | 3.00  (-4.80-10.54) |
| Total Cholesterol (mmol/L) | -0.84  (-1.27- -0.43) |
| LDL Cholesterol (mmol/L) | -0.55  (-0.98- -0.11) |
| HDL Cholesterol (mmol/L) | -0.08  (-0.21-0.05) |
| Triglyceride (mmol/L) | -0.66  (-0.88- -0.45) |
| Apo AI (g/L) | -0.20  (-0.29- -0.10) |
| Apo B (g/L) | -0.23  (-0.32- -0.14) |
| Apo CIII (mg/dL) | -4.27  (-5.59- -3.27) |
| Apo E (g/L) | -0.005  (-0.009- -0.001) |
